# Publisher Correction: Microwave photons emitted by fractionally charged quasiparticles

**DOI:** 10.1038/s41467-019-10199-9

**Published:** 2019-05-15

**Authors:** R. Bisognin, H. Bartolomei, M. Kumar, I. Safi, J.-M. Berroir, E. Bocquillon, B. Plaçais, A. Cavanna, U. Gennser, Y. Jin, G. Fève

**Affiliations:** 1 Laboratoire de Physique de l’ Ecole normale supérieure, ENS, Université PSL, CNRS, Sorbonne Université, Université Paris-Diderot, Sorbonne Paris Cité, 75005 Paris, France; 2 Laboratoire de Physique des Solides, Université Paris-Saclay, 91405 Orsay, France; 3 Centre de Nanosciences et de Nanotechnologies (C2N), CNRS, Univ. Paris Sud, Université Paris-Saclay, 91120 Palaiseau, France

**Keywords:** Electronic devices, Quantum Hall, Semiconductors

Correction to: *Nature Communications* 10.1038/s41467-019-09758-x, published online 12 April 2019

The original version of this Article contained an error in the author affiliations. Affiliation 3 incorrectly read “Centre de Nanosciences et de Nanotechnologies (C2N), CNRS, Univ. Paris Sud, Université Paris-Saclay, 91190, Saint-Aubin, France”. This has now been corrected in both the PDF and HTML versions of the Article.

This has now been corrected in both the PDF and HTML versions of the Article.

