# Supplementary material for: Microwave photons emitted by fractionally charged quasiparticles
Source: Nat Commun. 2019 Apr 12;10:1708. doi: 10.1038/s41467-019-09758-x (PMC6461615; doi:10.1038/s41467-019-09758-x)
Supplement: Supplementary file 1 — Supplementary Information [file 41467_2019_9758_MOESM1_ESM.pdf]

Supplementary Information for Microwave photons emitted by fractionally charged quasiparticles.

R. Bisognin[1], H. Bartolomei[1], M. Kumar[1], I. Safi[2], J.-M. Berroir[1], E. Bocquillon[1], B. Plaçais[1], A. Cavanna[3], U. Gennser[3], Y. Jin[3], and G. Fève[1*]

[1]Laboratoire de Physique de l'Ecole normale supérieure, ENS, Université PSL, CNRS, Sorbonne Université, Université Paris-Diderot, Sorbonne Paris Cité, Paris, France

[2] Laboratoire de Physique des Solides, Université Paris-Saclay, 91405 Orsay France

[3] Centre de Nanosciences et de Nanotechnologies (C2N), CNRS, Univ. Paris Sud, Université Paris-Saclay, 91120 Palaiseau, France.

[*] To whom correspondence should be addressed; E-mail: feve@lpa.ens.fr.

SUPPLEMENTARY NOTE 1. DETERMINATION OF THE FRACTIONAL CHARGE FROM LOW FREQUENCY MEASUREMENTS

A. $\nu = 4/3$

Supplementary Figure1.a represents both the differential conductance (red) through the QPC and the low frequency noise $\Delta S_{33}(f = 0)$ (blue) as a function of the QPC gate voltage for a fixed dc voltage $V_{dc} = -130\ \mu V$. The differential conductance shows a plateau for $G = e^2/h$ which is consistent with the following edge structure: two edge channels are successively transmitted with respective conductance $g_1 = e^2/h$ and $g_{1/3} = \frac{1}{3}e^2/h$. The noise measurements, $\Delta S_{33}(f = 0)$, plotted on Supplementary Figure1.b as a function of the QPC gate voltage support this picture. The noise takes its largest values when the $\nu = 1$ channel is partitioned ($0 \leq G \leq e^2/h$) with a qualitative agreement with a $q \approx e$ charge (dashed lines) although the agreement is not fully quantitative. The noise is suppressed when the $\nu = 1$ channel is perfectly transmitted ($G = e^2/h$) and increases again when the $\nu = 1/3$ channel is partitioned ($e^2/h \leq G \leq \frac{4}{3}e^2/h$). In this regime, we observe a quantitative agreement with the transfer of quasiparticles with charge $q = e/3$ represented by the dashed lines, defining the transmission of the $\nu = 1/3$ channel by $D = \big(G - e^2/h\big)/\big(e^2/(3h)\big)$. The noise does not vanish when the QPC gate voltage reaches its maximum value as we do not reach the perfect transmission of the $\nu = 1/3$ channel. Supplementary Figure1.c represents the bias voltage dependence of the noise for two transmissions of the $\nu = 1/3$ channel, $D \approx 0.65$ and $D \approx 0.4$. For the weakest strength of scattering ($D \approx 0.65$), the noise agrees quantitatively with the transfer of charge $q = e/3$ (blue dashed line). When the scattering strength is increased ($D \approx 0.4$), the low bias values of the noise correspond to the transfer of the charge $q = e$ (black dashed line). At higher values of the bias voltage, the noise falls back on the $q = e/3$ prediction (blue dashed line). This is consistent with the transition between a strong ($q = e$) to weak backscattering regime ($q = e/3$) when the bias is increased. For the high frequency noise measurements presented in the manuscript, we operate the QPC at transmission $D = 0.63$ corresponding to the transfer of fractional charges $q = e/3$.

B. $\nu = 2/3$

Supplementary Figure2.a represents our measurements of the low frequency noise at $\nu = 2/3$ as a function of the differential conductance (in units of $\frac{2}{3}e^2/h$) for almost all values of bias voltage

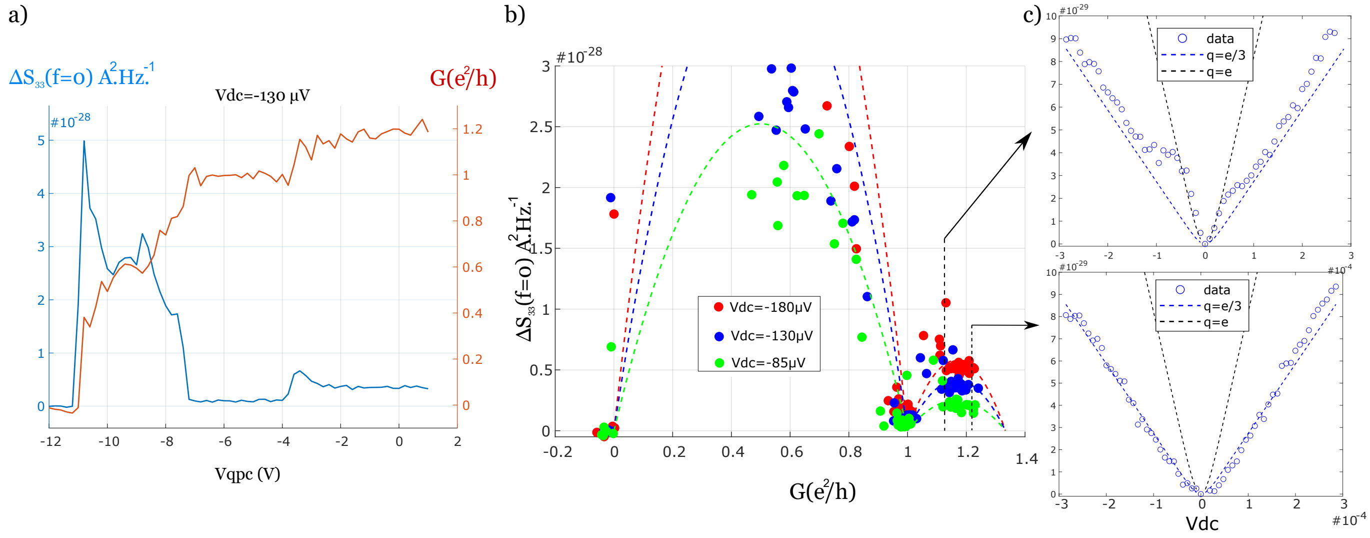

SUPP. FIG. 1. $\nu = 4/3$ **a.** Red: differential conductance through the QPC for $V_{dc} = -130\ \mu V$ as a function of the QPC gate voltage in units of e^2/h. We observe the successive transmission of a $\nu = 1$ channel of quantized conductance e^2/h followed by the transmission of a $\nu = 1/3$ channel which does not reach prefect transmission $(1/3e^2/h)$. Blue: Low frequency excess noise $\Delta S_{33}(f = 0, V_{dc} = -130\mu V)$ as a function of the QPC gate voltage. We observe a suppression of the noise at the $\nu = 1$ plateau followed by an increase of the noise when the $\nu = 1/3$ channel is partitioned. **b.** Low frequency noise $\Delta S_{33}(f = 0)$ as a function of the differential conductance for three values of the dc voltage $V_{dc} = -85\mu V$, $V_{dc} = -130\mu V$ and $V_{dc} = -180\mu V$. We observe the successive partitioning of two channels. The dashed lines represent the expected noise, using Eq.(1) of the main manuscript, with $q = e$ for $0 \leq G \leq e^2/h$ and $q = e/3$ for $e^2/h \leq G \leq \frac{4}{3}e^2/h$. **c.** Low frequency noise as a function of V_{dc} for two values of the transmission of the $\nu = 1/3$ channel: $D = 0.65$ (bottom) and $D = 0.4$ (top). The dashed line represent the expected noise from Eq.(1) of the main manuscript, with $q = e$ (black) and $q = e/3$ (blue).

$(90 \leq |V_{dc}| \leq 450\ \mu\text{V})$. In order to compare the noise values obtained at different bias voltages, we rescale the noise measurements by plotting $\Delta\tilde{S}_{33}(f = 0)$ defined by:

$$\Delta\tilde{S}_{33}(f = 0) = \frac{\Delta S_{33}(f = 0, V_{dc})}{2\frac{e}{3}\frac{2e^2}{3h}V_{dc}\big(\coth\big(\frac{eV_{dc}}{6k_BT}\big) - \frac{6k_BT}{eV_{dc}}\big)} \tag{1}$$

As can be seen on Supplementary Figure2.a, after the rescaling, the dependence in the dc bias is suppressed and all data for a given differential conductance fall on each other. Using the non-interacting formula (Eq.(1) of the main manuscript), the partitioning of a charge $q = e/3$ should then follow a $D(1 - D)$ dependence, where D is defined as the differential conductance in units

of $\frac{2}{3}\frac{e^2}{h}$. This is represented as the black dashed line on Supplementary Figure2.a which agrees very well with the data in the range $D \geq 0.6$. We also notice that the noise does not vanish for $D = 0.5$ which corresponds to a plateau in the conductance for $G = \frac{1}{3}\frac{e^2}{h}$. Finally, Supplementary Figure2.b represents the bias dependence of the noise in the weak backscattering regime, $D = 0.87$ (blue dots) and $D = 0.92$ (green dots). In this regime, the noise is consistent with the transfer of quasiparticles of charge $q = e/3$ (dashed lines) in the full range of dc bias.

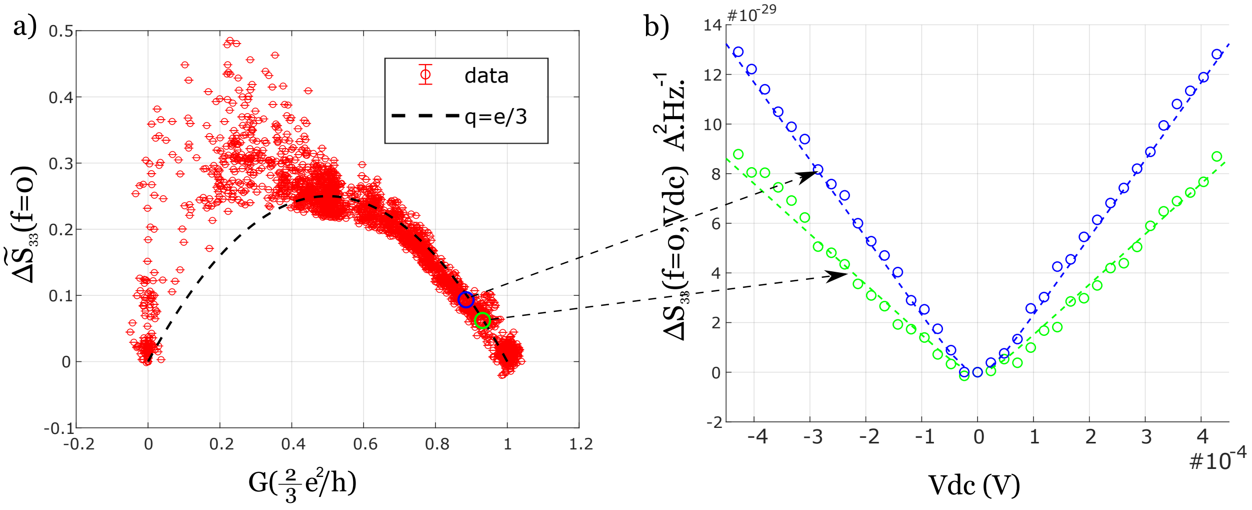

SUPP. FIG. 2. $\nu = 2/3$ **a.** Rescaled noise $\Delta\tilde{S}_{33}(f = 0)$ for all values of the bias voltage $(90 \leq |V_{dc}| \leq 450\ \mu\text{V})$ as a function of the transmission of the channel. For $D \geq 0.6$ the data is consistent with the transfer of the charge $q = e/3$ (black dashed line). **b.** $\Delta S_{33}(f = 0)$ as a function of the bias voltage for two values of the transmission $D = 0.87$ (blue dots) and $D = 0.92$ (green dots). For these transmissions corresponding to the weak backscattering limit, the noise values are consistent with the transfer of quasiparticles of charge $q = e/3$ (dashed lines).

SUPPLEMENTARY NOTE 2. NON-EQUILIBRIUM RELATION AND NON-LINEARITY OF THE BACKSCATTERED CURRENT FOR $\nu = 4/3$ AND $\nu = 2/3$

Our quantitative analysis of the noise values at high frequencies are based on non-equilibrium fluctuations relations that relate the high frequency noise of the backscattered current to its low frequency values through the characteristic frequency qV_{dc}/h. However, the noise of the backscattered current is not directly accessible and has to be connected to the noise at the output ports ΔS_{33} and ΔS_{44}. The relation between the high and low frequency values of ΔS_{ii} then involves a

correction δ which is related to the non-linearity of the backscattered current:

$$\Delta S_{44}(f, V_{dc}) = \frac{\Delta S_{44}^{sym}(0, V_{dc} + hf/q) + \Delta S_{44}^{sym}(0, V_{dc} - hf/q)}{2} - \delta \tag{2}$$

$$\delta = c(f) - c(f = 0) \tag{3}$$

$$c(f) = q \coth\left(\frac{hf}{2k_B T}\right)\frac{I_b(V_{dc} + hf/q) - I_b(V_{dc} - hf/q)}{2} \tag{4}$$

By measuring the differential conductance, we can deduce the bias dependence of the backscattered current and evaluate the correction δ. The backscattered currents measured for the transmissions discussed in the main text are plotted on Supplementary Figure3.a ($\nu = 4/3$) and Supplementary Figure4.a ($\nu = 2/3$). In both cases, for these values of the transmission, the measured non-linearity is small, such that the correction δ is also expected to be small. Evaluations of δ are plotted on Supplementary Figure3.b and Supplementary Figure4.b together with the calculations of $\Delta S_{44}(f, V_{dc})$ using Eq.(2) where we assume $\delta = 0$. For both filling factors the correction δ is always smaller than 5% of $\Delta S_{44}(f, V_{dc})$ which justifies the assumption made in the manuscript: $\delta \approx 0$.

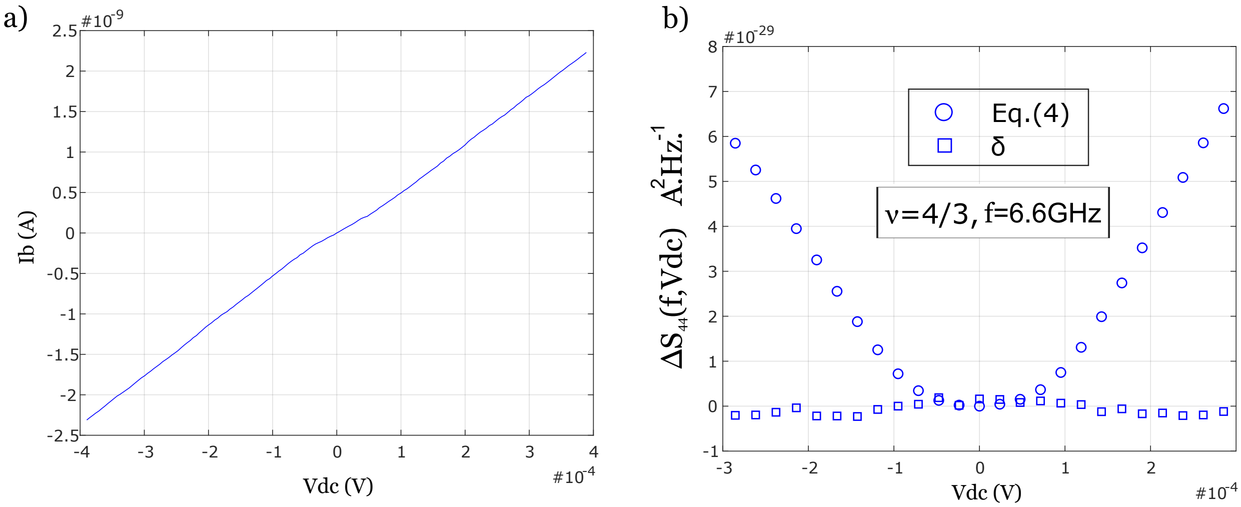

SUPP. FIG. 3. Backscattered current and correction δ for $\nu = 4/3$. **a.** Backscattered current $I_b(V_{dc})$. **b.** Numerical evaluation of δ (square) using Eqs.(3) and (4) and of $\Delta S_{44}(f, V_{dc})$ (dots) using Eq.(2) with $\delta = 0$.

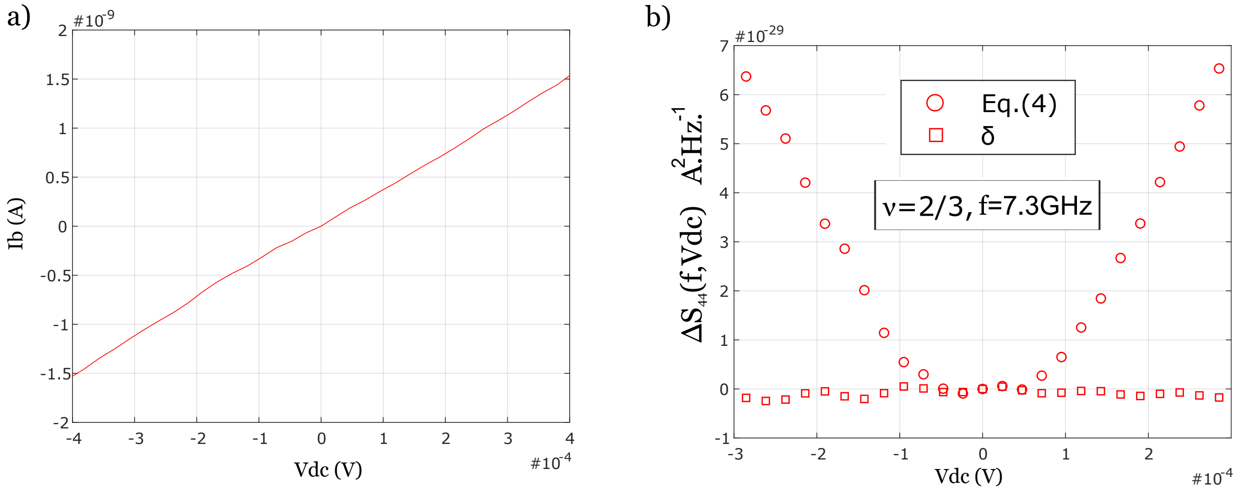

SUPP. FIG. 4. Backscattered current and correction δ for $\nu = 2/3$. **a.** Backscattered current $I_b(V_{dc})$. **b.** Numerical evaluation of δ (square) using Eqs.(3) and (4) and of $\Delta S_{44}(f, V_{dc})$ (dots) using Eq.(2) with $\delta = 0$.

SUPPLEMENTARY NOTE 3. ADDITIONAL HIGH FREQUENCY NOISE MEASUREMENTS AT $\nu = 3$ AND $\nu = 4/3$

Figure4.b of the main manuscript presents all the measured thresholds V_0 for various filling factor and measurement frequencies f. These additional measurements realized for $\nu = 3$ ($f = 9.5$ GHz), and $\nu = 4/3$ ($f = 3.5$ and $f = 8$ GHz), are plotted on Supplementary Figure5 and Supplementary Figure6. At $\nu = 4/3$, the signal over noise ratio for these measurements is not as good as the measurement presented in the main manuscript ($f = 6.6$ GHz). Indeed the measurement frequencies $f = 3.5$ and $f = 8$ GHz fall at the edges of the measurement bandwidth imposed by the circulators placed between the cryogenic amplifiers and the sample. This explains the larger size of the error bars (the noise data for $f = 8$ GHz are averaged between positive and negative bias voltage V_{dc} to reduce the size of error bars). Consequently, the thresholds V_0 deduced from numerical fits of the data using Eq.(2) of the main manuscript have larger error bars. For $\nu = 3$ the signal over noise ratio is much larger (due to larger conductance of the channel and a larger transferred charge $q = e$). Consequently, the extraction of the threshold is more accurate.

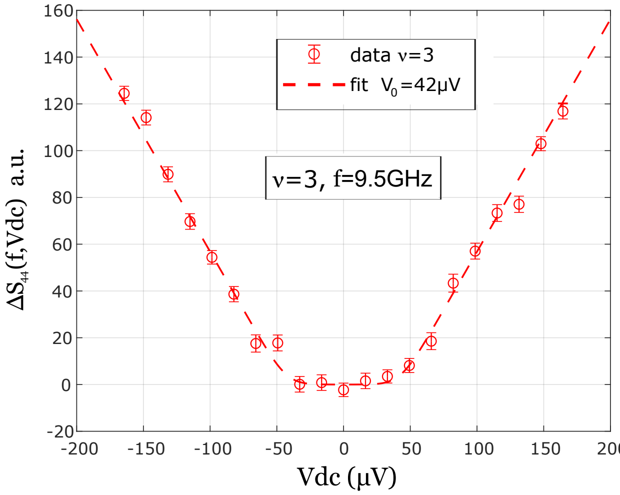

SUPP. FIG. 5. High frequency measurement, $\nu = 3$, $f = 9.5$ GHz. Data points are represented by red dots, dashed lines represent the fit by Eq.(2) of the main manuscript with $V_0 = 42.8 \pm 2.6\ \mu V$. Error bars are defined as standard error of the mean.

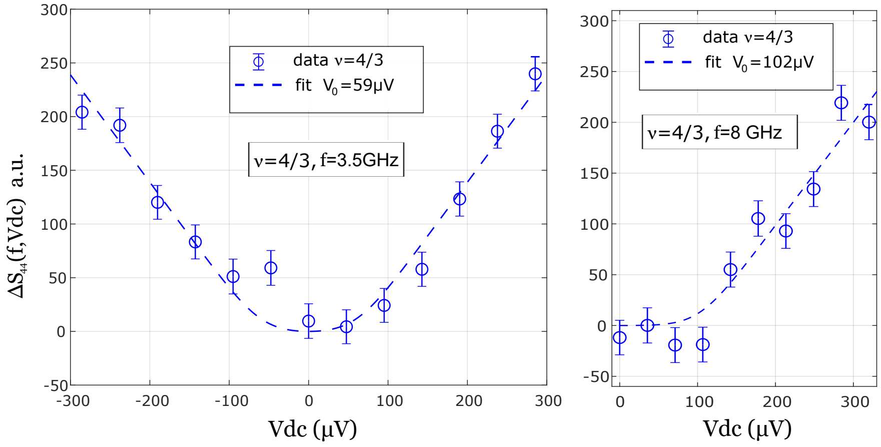

SUPP. FIG. 6. High frequency measurement, $\nu = 3$, $f = 3.5$ and $f = 8$ GHz. Data points are represented by blue dots, dashed lines represent the fit by Eq.(2) of the main manuscript with $V_0 = 59 \pm 17\ \mu V$ ($f = 3.5$ GHz) and $V_0 = 102 \pm 23\ \mu V$ ($f = 8$ GHz) . Error bars are defined as standard error of the mean.
